# Comparison of Shear Bond Strength and Estimation of Adhesive Remnant Index between Light-cure Composite and Dual-cure Composite: An *in vitro* Study

**DOI:** 10.5005/jp-journals-10005-1212

**Published:** 2013-10-14

**Authors:** Geeta Verma, Mridula Trehan, Sunil Sharma

**Affiliations:** Postgraduate Student (III year), Department of Orthodontics and Dentofacial Orthopedics, Mahatma Gandhi Dental College and Hospital, Jaipur, Rajasthan, India, Phone: 9828510491, e-mail: dr.geetavema@yahoo.in; Professor and Head, Department of Orthodontics, Mahatma Gandhi Dental College and Hospital, Jaipur, Rajasthan, India; Professor and Head, Department of Oral and Maxillofacial Surgery Mahatma Gandhi Dental College and Hospital, Jaipur, Rajasthan India

**Keywords:** Light-cure, Dual-cure, Shear bond strength, Adhesive remnant index

## Abstract

**Aims and objectives:** To measure and compare the shear bond strength and adhesive remnant index of light-cure composite. (Enlight, Ormco.) and dual-cure composite (Phase II dual cure, Reliance Ortho).

**Materials and methods:** Sixty extracted human premolar teeth were divided into two groups: group I (blue): conventional light cure composite resin. (Enlight, Ormco.) and group II (green): dual cure composite resin. (Phase II dual cure, Reliance Ortho.) with 30 teeth in each group. These samples were tested on the universal testing machine to measure the shear bond strength.

**Results:** Student t-test showed that the mean shear bond strength of the conventional light cure group (8.54 MPa - 10.42 MPa) was significantly lower than dual cure group (10.45 MPa -12.17 MPa).

**Conclusion:** These findings indicate that the shear bond strength of dual-cure composite resin (Phase II dual cure, Reliance Ortho) is comparatively higher than conventional light-cure composite resin (Enlight, Ormco). In the majority of the samples, adhesive remnant index (ARI) scores were 4 and 5 in both the groups whereas score 1 is attained by the least number of samples in both the groups.

**How to cite this article:** Verma G, Trehan M, Sharma S. Comparison of Shear Bond Strength and Estimation of Adhesive Remnant Index between Light-cure Composite and Dual-cure Composite: An *in vitro* Study. Int J Clin Pediatr Dent 2013;6(3):166-170.

## INTRODUCTION

Rapid strides in material science over the years have produced progressively advanced materials making the direct bonding procedure more precise, comfortable and time-effective. By the late 1970s bonding of orthodontic brackets had become an accepted clinical technique in routine fixed appliance treatment. Self-cure resin provides good bond strength, it has a few inherent flaws like being extremely technique sensitive, having a short setting time which affects bracket positioning accuracy and low initial bond strength.

In 1978 after their introduction, light activated composites have largely replaced chemically activated systems in dentistry. This has been due to the clinical advantages of these products in that it gives the operator virtually unlimited working time to position the brackets accurately since the material can be cured at will. It is easier to remove excess resin before setting is initiated. Light activated composites have a higher initial bond strength enabling immediate placement of archwires. In spite of these advantages the main disadvantages of light activated composites are the increased time for bonding and the clinician may never be totally assured of the complete polymerization of the resin under the bracket.^1-8^

Incomplete polymerized areas within the adhesive layer of the resin cement may allow for the diffusion of water that may impair the bond strength in these areas and thus compromise the long-term effectiveness of the adhesive resin cement. So, to overcome these drawbacks as well as to directly control the setting time of the resin cement and to improve the polymerization conversion of the resin cement that exists under the metal bracket, a dual-curing type composite resin cement, designed with additional capabilities, e.g. self-adhesiveness and fluoride release was introduced.^9,10^

Dual-cure composite resin cement consists of two types of pastes, a catalyst paste and a base paste. In these systems, activation of polymerization is induced through surface exposure of the material to the source of visible light, and polymerization in the bulk material occurs by a chemical curing process. In a study , the dual cure adhesive was found to provide significantly higher bond strength compared to chemically cured and light cured materials 24 hours following activation.^5,11-13^

Very few studies have been conducted on the properties of dual cure composites. So the purpose of my research is to compare the shear bond strength and to assess adhesive remnant index (ARI) between dual cure composite and conventional light cure composite.

## AIMS AND OBJECTIVES

The aims and objectives of this study are as follows:

- To measure and compare the shear bond strength of orthodontic stainless steel brackets bonded to enamel using–light-cure composite resin (Enlight, Ormco) and dual-cure composite resin (Phase II dual cure, Reliance Ortho.).
- Estimation and comparison of ARI using - light cure composite resin (Enlight, Ormco) and dual cure composite resin. (Phase II dual cure, Reliance Ortho.).

## MATERIALS AND METHODS

Sixty freshly extracted human maxillary and mandibular premolar teeth were collected for the study. Selected teeth were free of enamel decalcification, caries, cracks on labial surface, or fluorosis and were not subjected to any pretreatment chemical agents, e.g. hydrogen peroxide. Ethical clearance has been obtained.

External surfaces of all the teeth were thoroughly cleaned with ultrasonic scaler to remove tissue tags and plaque. They were stored in distillled water at room temperature till use. All the extracted teeth were used within 6 months of extraction.

### Mounting of Premolars

**Fig. 1:**
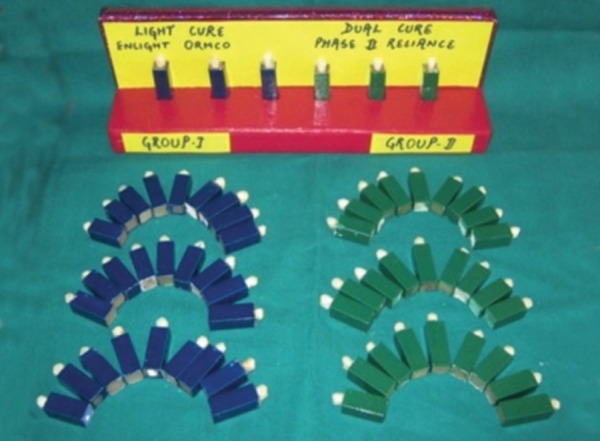
Mounted premolars divided in to two groups and color coded

Selected premolars were embedded in Aluminum cubes of 2.5 × 1 × 1 cm^3^ with the help of self-cure acrylic so as to prevent any displacement of teeth during shear bond strength testing. During mounting, care was taken so as to align the facial surfaces of the teeth perpendicular with the bottom of the cube so that the labial surface would be parallel to the applied force during the shear test. The mounted premolars were randomly divided into two groups, each group containing 30 teeth. All the samples were color coded as per the colors allocated to the groups (Fig. 1).

*Group I (blue):* Conventional light cure composite resin (Enlight, Ormco.)

*Group II (green):* Dual cure composite resin (Phase II dual cure, Reliance Ortho.

### Bonding of the Brackets

Teeth were etched with Ezee etch – 37, etchent gel for 30 seconds, then rinsed with a water spray. The Primer (Ortho Solo, Ormco) was applied on the etched surfaces and light cured for 20 seconds. The adhesive (Enlight Light Cure, Ormco) was placed on each bracket base. The bracket was placed firmly on the tooth to desired position and angulation and were then light cured for 40 seconds. The samples were stored in water at 37°C for 24 hours in an incubator, till bond strength testing. In case of dual cure composite resin.(Phase II dual cure, Reliance Ortho.) Enamel conditioning and sealant application was done in the same way as light cure. The bonding paste was immediately prepared by placing equal portions of Paste A and B onto the mixing pad and the two pastes were spatulated for a maximum of 10 seconds till a homogenous mixture was obtained. A one-to-one mix ratio of catalyst to base produces a 4 minute working time from the start of mix. The mixed paste was shielded from intense ambient light. The mixed paste was applied to the bracket base with a wooden applicator in a sufficient volume so as to cover the bracket base fully before placement on the tooth. Bracket is positioned and cured in the same way as light cure.

### Shear Bond Strength Testing

**Fig. 2:**
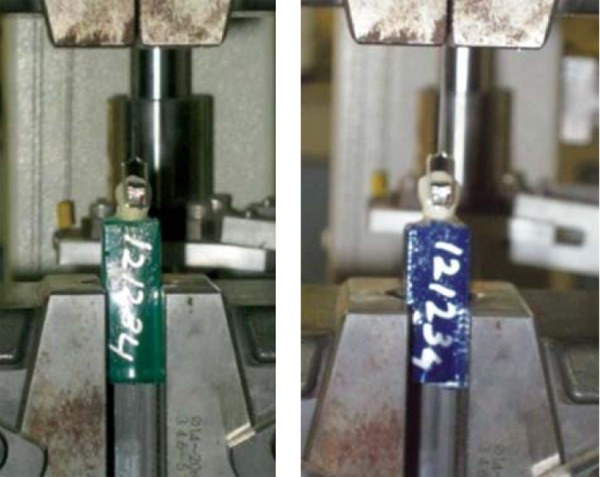
Aluminum cubes placed in lower clamp (Fixed Head) of the universal testing machine

The teeth were debonded after 24 hours from the time of initial bonding using Schimadzu computer controlled universal testing machine (UTM). A rectangular stainless steel wire (0.017 × 0.025) loop was used to apply occlusogingival load for shear bond strength testing. The occlusogingival load was applied at bracket base-resin interface with a cross head speed of 1mm/min (Fig. 2). The force producing failure was recorded in Newtons. The surface area of the bracket base was calculated to be 11.00/mm^2^, with the help of digital vernier's callipers. While conducting the testing, none of the enamel suface or the tooth fractured. The bond strength was calculated in MegaPascals by using the following formula:^14^

    Bond strength in MPa = Force in Newton/Surface area of bracket in mm^2^    

## ASSESSMENT OF ADHESIVE REMNANT INDEX

**Fig. 3:**
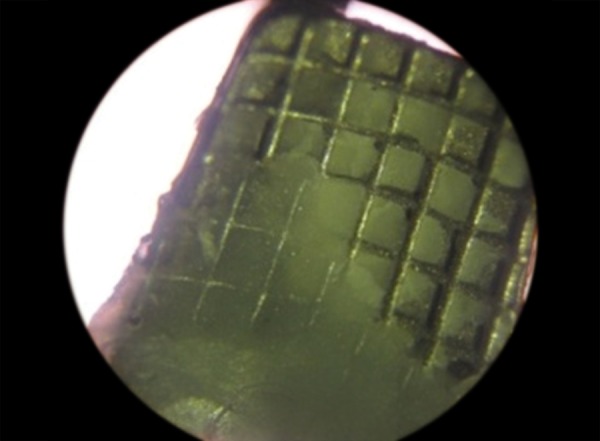
Adhesive remnant on bracket surface

Debonded brackets were seen under simple microscope using 15× magnification (Fig. 3). The mode of bond failure was assessed by the percentage of the number of mesh gauze occupied by the adhesive remaining on the bracket base after debonding devided by the total number of mesh gauze of the bracket base. Formula for its calculation is as follows:

    ARI = (Numberof mesh gauze occupied by the adhesive/Total number of mesh gauze of the bracket base) × 100    

Later, each tooth was assigned a modified ARI^15^ value according to the following criteria:

Score 1    =   All of the composite remained on the tooth, with an impression of the bracket base.

Score 2    =   More than 90% of the composite remained on the tooth.

Score 3    =   More than 10% but less than 90% of the composite remained on the tooth.

Score 4    =   Less than 10% of composite remained on the tooth surface.

Score 5    =   No composite remained on the enamel.

### Statistical Analysis

The shear bond strength of each sample was measured Student t-test was used to determine whether significan differences were present in the bond strength between the two groups, i.e. Conventional light-cure composite resin. Enlight, Ormco (blue colored) and dual cure composite resin. Phase II dual cure, Reliance Ortho (green colored).

Pearson Chi-square test was applied to calculate the ARI among the two groups.

## RESULTS

### Shear Bond Strength

**Graph 1:**
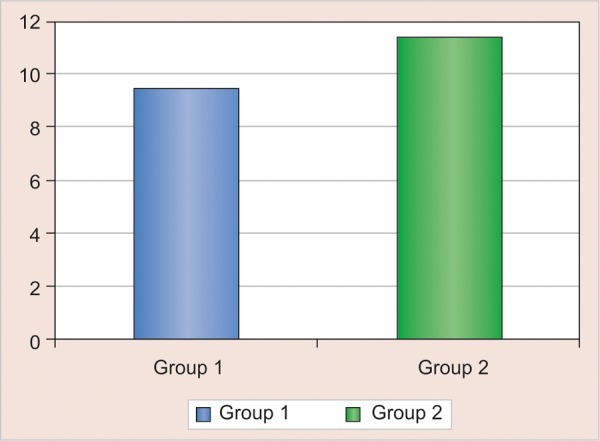
Bar diagram representating the comparison of mean shear bond strength in MPa of two groups

In the conventional light-cure group, the shear bond strength ranged from 8.54 to 10.42 MPa and in case of dual-cure group, the shear bond strength ranged from 10.45 to 12.17 MPa (Table 1). This shows that the shear bond strength of the dual cure group was significantly higher than conventional light-cure group (Graph 1).

### Adhesive Remnant Index

In our study most of the samples of both the groups, i.e. light-cure ( Table 2) as well as dual-cure ( Table 3) achieved scores 4 and 5 (more than 90% or all of the adhesive remained on the bracket) with a very low frequency of ARI scores 1 and 2 (more than 90% or all of the adhesive remained on the enamel).

**Table 1:** **Table 1:** Shear bond strength between two groups

*Groups*	*N*		*Mean*	*SD*	*SEM*
Group I	30		9.484	0.5438	0.09929
Group II	30		11.42	0.4923	0.08988
		*Value*	*Degree of freedom_(df)_*	*p-value*	HS/NS/S
Student t-test		-14.438	58	0.000	HS

**Table 2:** **Table 2:** ARI scores of group I (bonded with Enlight Ormco)

*Scores*		*No. of samples*		*Percentage*
Score = 1		2		7
Score = 2		1		3
Score = 3		6		20
Score = 4		10		33
Score = 5		11		37

**Table 3:** **Table 3:** ARI scores of group II (bonded with phase II dual cure)

*Scores*		*No. of samples*		*Percentage*
Score = 1		1		3
Score = 2		3		10
Score = 3		2		7
Score = 4		11		37
Score = 5		13		43

## DISCUSSION

Ever since the bonding procedure was introduced by Newman**^7^** into orthodontic practice, there has been a constant endeavor to improve the qualities of bonding materials. The search still continues. The advantages of direct bonding include benefit for both the patient and the practitioner. For patients, there is less risk of enamel decalcification, better oral hygiene maintenance, decreased irritation of the gingival tissue and improved esthetics. For practitioners, direct bonding eliminates pretreatment separation of the teeth and decreases the chairside time.^3,4^

Ideally, the bond strength needs to be optimum rather than too much or too less. Excessive bond strength increases the risk of enamel damage during debonding, and too weak bond strength results in frequent bond failures during the course of treatment. According to Reynolds IR.^9^ the optimum bond strength should be in the range of 6 to 8 MPa.Rapid strides in material science over the years have produced progressively advanced materials making the direct bonding procedure more precise, comfortable and time-effective.

Our study basically aims to measure the shear bond strength of orthodontic brackets, using light-cure composite resin (Enlight, Ormco) and dual-cure composite resin (Phase II dual cure, Reliance Ortho), so that we get a better idea about the clinical performance of this new material when compared simultaneously with the conventional one.

Student t-test showed that the mean shear bond strength of the conventional light cure group (8.54 - 10.42 MPa) was significantly lower than dual cure group (10.45 - 12.17 MPa). Pearson Chi-square test for ARI showed nonsignificant difference between the two groups. These findings indicate that conventional light cure group provides stronger shear bond strength as compared to dual cure group.

These findings are similar to those of Smith RT and Shivapuja PK (1993)^16^, Newman GV, Sun BC, Ozsoylu SA, Newman RA (1994)^17^, Sargison AE, McCabe JF, Gordon PH (1995)^18^, Kasuya K, Miyazaki Y, Ogawa N, Maki K,

Manabe A, Itoh K, et al (2006)^19^, Evgenija M, Branislav G, Ivana S, Dejan M, Vukoman J et al (2008).^5^

Adhesive remnant of debonded brackets are seen under 15× magnification using simple microscope and scored using modified ARI. Initially ARI was given by Årtun J, Bergland S (1984).^20^ Later on Bishara SE, Trulove TS (1990)^15^ used modified ARI.

In our study most of the samples of both the groups, i.e. light-cure as well as dual-cure achieved scores 4 and 5 (more than 9 0% or all of the adhesive remained on the btracket) with a very low freduency of ARI scores 1 and 2 (more than 90% or all of the adhesive remained on the enamel). This suggests that the bond to the bracket is stronger than the bond to the enamel. This is in agreement with previous studies done by McSherry PF (1996),^21^ Imad Shammaa et al (1999),^22^ Summers A, Kao E, Gilmore J, Gunel E, Ngan P (2004),^23^ Al Shamsi A, Cunningham J, Lamey PJ, Lynch E (2006),^1^ Ritter AV, Ghanam E, Luiz AF (2009).^24^

Since, the bond failure occurs at the adhesive-enamel interface, it is easier for clinician to clean up the adhesive on the enamel surface after debonding because the removal of remnant adhesive from the tooth surface may lead to enamel damage and may increase chairside time.

A study by O'Brien KD, Watts DC and Read MJF (1988)^25^ suggested that the ARI score depended on many factors, which included the bracket base design and the adhesive type, and not only the bond strengths at the interfaces. Retief DH, Dreyer CJ, Gavron G (1970)^26^reported that enamel failure occurred when the bond strength exceeded 13.5 MPa. The results of our study agreed with this observation in that the enamel fracture occurred at higher stress.

Via this study a clearer picture about the shear bond strength of the newer dual cure and the conventional adhesive materials is obtained. As both these groups were compared together, this study accurately tells us about the clinical performances of these two materials, and also gives us an insight on how they can be utilized in different clinical situations.

This shows that these two materials are sufficiently efficient for reliable bonding, this newer dual-cure material add up to the armamentarium of the orthodontist and can be used judiciously in different clinical situations.

## CONCLUSION

The present study was an *in vitro* study designed to test the shear bond strength and estimation of ARI of orthodontic brackets bonded to enamel using–light cure composite resin (Enlight, Ormco) and dual cure composite resin (Phase II dual cure, Reliance Ortho).

The following conclusions can be drawn from the study:

- The shear bond strength of dual-cure composite resin (Phase II dual cure, Reliance Ortho) is comparatively higher than conventional light-cure composite resin (Enlight, Ormco); but is definitely more or similar to the required bond strength to resist masticatory forces.
- ARI showed that most of the bond failures in both groups were between the tooth surface and adhesive whereas in very small amount adhesive remnant left on enamel surface.

Keeping these factors in mind, the use of dual-cure composite resin can be recommended as an alternative method for bonding orthodontic brackets.
